# Antibiotics With Percutaneous Aspiration or Drainage for Pyogenic Liver Abscess

**DOI:** 10.1155/1993/71698

**Published:** 1993

**Authors:** D. D'Amico

**Affiliations:** Universita Degli Studi di Padova Via Giustianini 2 Padova 35128 Italy


*HPB Surgery*, 1993, Vol. 6, pp. 325–333
Reprints available directly from the publisher
Photocopying permitted by license only

© 1993 Harwood Academic Publishers GmbH
Printed in the United States of America

### HPB INTERNATIONAL

**EDITORIAL & ABSTRACTING SERVICE**

JOHN TERBLANCHE, EDITOR
Department of Surgery,
Medical School · Observatory 7925
Cape Town · South Africa
Telephone: (021) 47-1250 Ext. 2
Telefax (021) 448-6461

### ANTIBIOTICS WITH PERCUTANEOUS ASPIRATION OR DRAINAGE FOR PYOGENIC LIVER ABSCESS

### ABSTRACT

*Stain, S.C., Yellin, A.E., Donovan, A.J. and Brien, H.W. Pyogenic Liver Abscess. Archives of Surgery, 126: 991–996.*

**Historically, open surgical drainage has been the treatment of choice for pyogenic liver abscess. The records of 54 patients with pyogenic liver abscess were reviewed to determine whether earlier diagnosis with current imaging tests and definitive treatment with antibiotics, aspiration, or catheter drainage was an effective alternative to open drainage. Twenty-nine patients were treated with broad-spectrum antibiotics and diagnostic aspiration. Twenty-three (79%) recovered uneventfully, and six required catheter or operative drainage. Twenty-three patients (including five who failed aspiration) underwent drainage with percutaneously placed catheters. Nineteen (83%) recovered; four required open surgical drainage. Of seven patients who required open surgical drainage, six recovered. One (2%) of the 54 patients died following failed aspiration and catheter and surgical drainage. Four patients were successfully treated with antibiotics alone without aspiration. These results confirm that pyogenic liver abscess can be successfully treated with broad-spectrum antibiotics and aspiration or percutaneous catheter drainage. Open surgical drainage is reserved for patients in whom treatment fails or who require celiotomy for concurrent disease.**

### PAPER DISCUSSION

KEYWORDS: Liver abscess

The methods of diagnosis and treatment of pyogenic liver abscess have surely undergone considerable changes in the past ten years.

As a matter of fact, advances in imaging techniques, including ultrasonography and computed tomography, may provide a prompt diagnosis with an accuracy that approaches 100%, ranging from 85 to 100% in the various series, and mortality rates have decreased consistently from the early 77%–95%, as first reported by Ochsner and De Bakey in 1938,[1] to the current figures of 19.2%[2] and 16%–18%[3] down to 11%[4].

Therapeutic options have changed likewise over the years, lessening the role for surgical intervention in selected cases, with the majority of pyogenic liver abscesses being nowadays treated by percutaneous drainage or needle aspiration. The paper from Los Angeles by a group with published previous experience is a thorough outline of the modern aspects concerning this disease starting from the microbiologic findings which reveal an ever increasing prevalence of polymicrobial and anaerobic infections as a causative factor. This point is widely supported by recent literature. The paper includes 29 patients. Needle aspiration and specific antibiotic therapy, based upon the bacteriologic results, was employed for abscesses of different size, varying from 2 to 15 cm in diameter: about 20% of these patients required subsequent treatment, either percutaneous or surgical. They do not state in which patients the primary treatment failed, what the size of the abscess was, the condition of the patients before undergoing treatment, what the underlying disease responsible for the liver abscess was and finally, in how many patients the hepatic lesion was solitary.

Another point that I would like to focus upon is the cost-benefit ratio considering that the duration of hospitalization for those patients ranged from six to forty-nine days.

The therapeutic effectiveness of percutaneous drainage is well-known as well as the complications and the length of hospitalization related to this procedure. However, it is worthwhile to mention the experience reported by the group from Duke University who described a recurrence rate of about 41% within 2 weeks after percutaneous drainage of liver abscesses while it was only 19% when the abscess was drained surgically.

The choice of surgical approach for the drainage of hepatic abscess has become the major subject of controversy and surgery is commonly regarded as a secondary treatment, as also emphasized in this article. The high mortality rate of up to 32%, reported for open surgical drainage is likely due to the the fact that the patients undergoing surgery usually have failed a previous percutaneous drainage or, alternatively, require operation for concurrent abdominal disease.

On the other hand, the data reported from Duke University, showed a mortality rate of 45% with antibiotic therapy alone, 25% following percutaneous drainage and 9.5% following surgical drainage. These features are strikingly in contrast to the data presented by the authors of this article.

It is difficult to compare the merits of different procedures mainly because the data obtained usually refer to experiences that seldom overlap.

In my opinion the guidelines that have been suggested in this paper do not apply to every patient with a liver abscess and a more individualized approach would seem more appropriate.

I, therefore, believe that exploratory needle aspiration and subsequent antibiotic therapy should be reserved for those patients with a single, small-sized abscess, without underlying abdominal pathology. As far as percutaneous drainage is concerned, it certainly is a reliable procedure by which pus and necrotic debris may be removed and the abscess cavity washed. Nevertheless I do not believe that it will be of any help in treating multiple, superficially located abscesses or in the management of patients with severe sepsis and associated pathology such as in acute suppurative cholangitis where the obstruction of the biliary tract results in multiple abscesses scattered throughout both hepatic lobes and severe sepsis occurs accounting for the high mortality rate.

The most correct therapeutic approach to pyogenic liver abscess requires an accurate diagnostic work-up aimed at precisely defining size, location and the number of lesions as well as the type of pathogens involved. In dealing with this desease, one last point deserves to be mentioned, the basic rule from times long past, which still holds good: *Ubi pus ibi evacuat.*

### REFERENCES

1. Ochsner, A., De Bakey, M. and Murray, S. (1938) Pyogenic abscess of the Liver. *Am. J. Surg.*, **40**, 292–314
2. King-Teh Lee, Pai-Ching Sheen, John-Shyong Chen, Chen-Guo Ker (1991) Pyogenic liver abscess: multivariate analysis of risk factors. *World J. Surg.*, **15**, 372–377
3. Branum, G.D., Tyson, G.S., Branum, M.A. and Meyers, W.C. (1990) Hepatic abscess: changes in etiology, diagnosis and management. *Ann. Surg.*, 212, **6**, 655–662
4. Bissada, A.A. and Bateman, J. (1991) Pyogenic liver abscess: a 7-year experience in a large Community Hospital. *Hepato-Gastroent*, **38**, 317–319

Professor D. D'Amico
Universita Degli Studi di Padova
Via Giustianini 2
35128 Padova
Italy

### PROPHYLACTIC SCLEROTHERAPY FOR OESOPHAGEAL VARICES

### ABSTRACT

*The Veterans Affairs Cooperative Variceal Sclerotherapy Group. (1991) Prophylactic sclerotherapy for esophageal varices in men with alcoholic liver disease. The New England Journal of Medicine;* **324**: 1779–1784.

***Background.*** **Sclerotherapy is an effective treatment for bleeding esophageal varices in patients with alcoholic liver disease. It has also been suggested that sclerotherapy might be effective in preventing initial episodes of bleeding and improving survival among such patients.**

